# Transcriptome data for *Hevea brasiliensis* associated with powdery Mildew infection

**DOI:** 10.1016/j.dib.2022.108254

**Published:** 2022-05-11

**Authors:** Urwashi Kamerkar, Ahmad Sofiman Othman

**Affiliations:** a School of Biological Sciences, Universiti Sains Malaysia, 11800 Minden, Pulau Pinang, Malaysia; b Centre of Chemical Biology, Universiti Sains Malaysia, 11900 Bayan Lepas, Pulau Pinang, Malaysia

**Keywords:** Transcriptomics, RNA-Seq, *Oidium heveae*, *Hevea brasiliensis*, Pathogen resistance

## Abstract

The *Hevea brasiliensis* or rubber tree belongs to the Euphorbiaceae family and is the only economically viable natural rubber source worldwide. The development of enhanced rubber tree clones with agronomically important traits is critical due to the growing demand for natural rubber around the world. Throughout the years, numerous disease-causing pathogens of *H. brasiliensis* have been identified and studied. One of the more prominent diseases affecting *H. brasiliensis* is powdery mildew caused by *Oidium heveae. Oidium heveae* primarily infects the newly formed leaves and buds of *H. brasiliensis*. Severe *Oidium heveae* infections cause extensive defoliation and yield loss. We performed RNA sequencing (RNA-Seq) for healthy and *O. heveae*-infected leaf tissues from RRIM 2025 and RRIM 929 rubber tree clones using the Illumina HiSeq 2000 platform. RNA-Seq generated 92007684 (12.9 GB) and 96070286 (13.5 GB) paired raw reads for healthy *H. brasiliensis* clones RRIM 2025 and RRIM 929 respectively. Similarly, RNA-Seq generated 93747858 (13.2 GB) and 93324564 (13.1 GB) paired raw reads for disease-infected *H. brasiliensis* clones RRIM 2025 and RRIM 929 respectively. The raw data were deposited in the NCBI under bio-project accession number PRJNA723431. The raw reads were quality trimmed and the reference-based transcriptome assembly was generated using the *H. brasiliensis* genome (ASM165405v1). The data were used to identify between the significantly differentially expressed genes of the healthy and diseased samples.

## Specifications Table

**Table:** 

Subject	Biological sciences
Specific subject area	Omics: transcriptomics
Type of data	Table, figure
How data were acquired	RNA sequencing data generated using the Illumina HiSeq. 2000 platform
Data format	Raw and analysed
Description of data collection	Total RNA was extracted from leaf of control (healthy) and powdery mildew-infected *H. brasiliensis* clones (RRIM 2025 and RRIM 929) and was sequenced using Illumina HiSeq. 2000 platform.
Data source location	City/town/region: Liman Plantation, Kedah Country: Malaysia
Data accessibility	The bio project and raw reads are available in the National Center for Biotechnology Information database Respository name: NCBI Data Identification Number: PRJNA723431 Direct URL to raw data: https://www.ncbi.nlm.nih.gov/bioproject/PRJNA723431 Supplementary files such as upregulated and downregulated genes in the two rubber clones are available in Mendeley Data Respository name: Mendeley Data Data Identification Number: DOI 10.17632/2mcfj7hw2y Direct URL to raw data: https://data.mendeley.com/datasets/2mcfj7hw2y/1

## Value of the Data

• Transcriptome data generated from the leaves of plants infected with powdery mildew could provide information on the molecular mechanism related to disease tolerance
• The differential expression analysis of controlled and diseased *H. brasiliensis* plants could compare the expression variability of the genes and subsequently help identify the pathogen response genes expressed specifically during pathogen invasion
• The information obtained from these data will provide a baseline understanding of the genes of practical importance in a resistance breeding programme.

## 1 Data Description

**Fig. 1:**
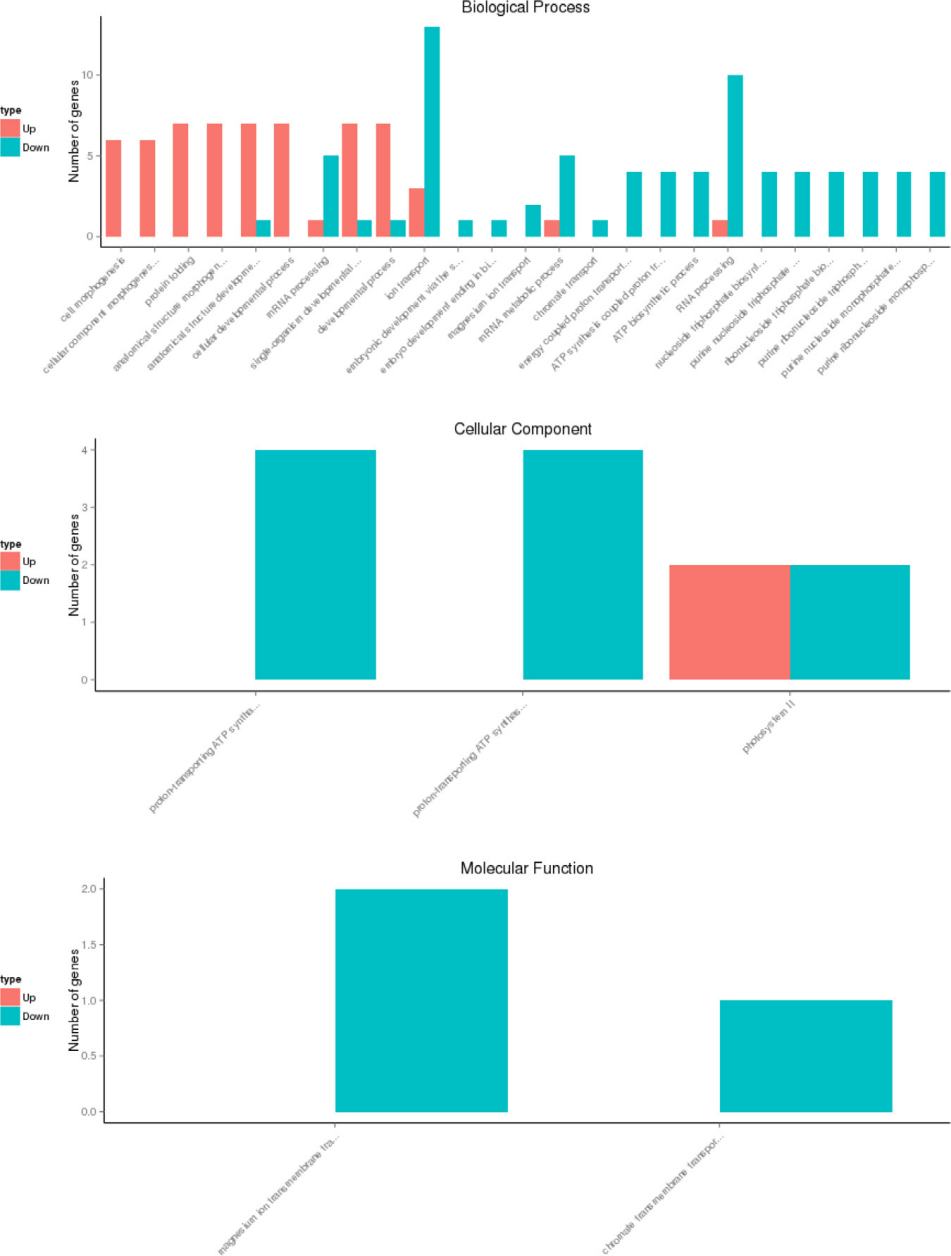
GO term enrichment analysis for DEG identified in RRIM 929 The up-regulated genes are represented in orange and the down-regulated genes are represented in blue.

**Fig. 2:**
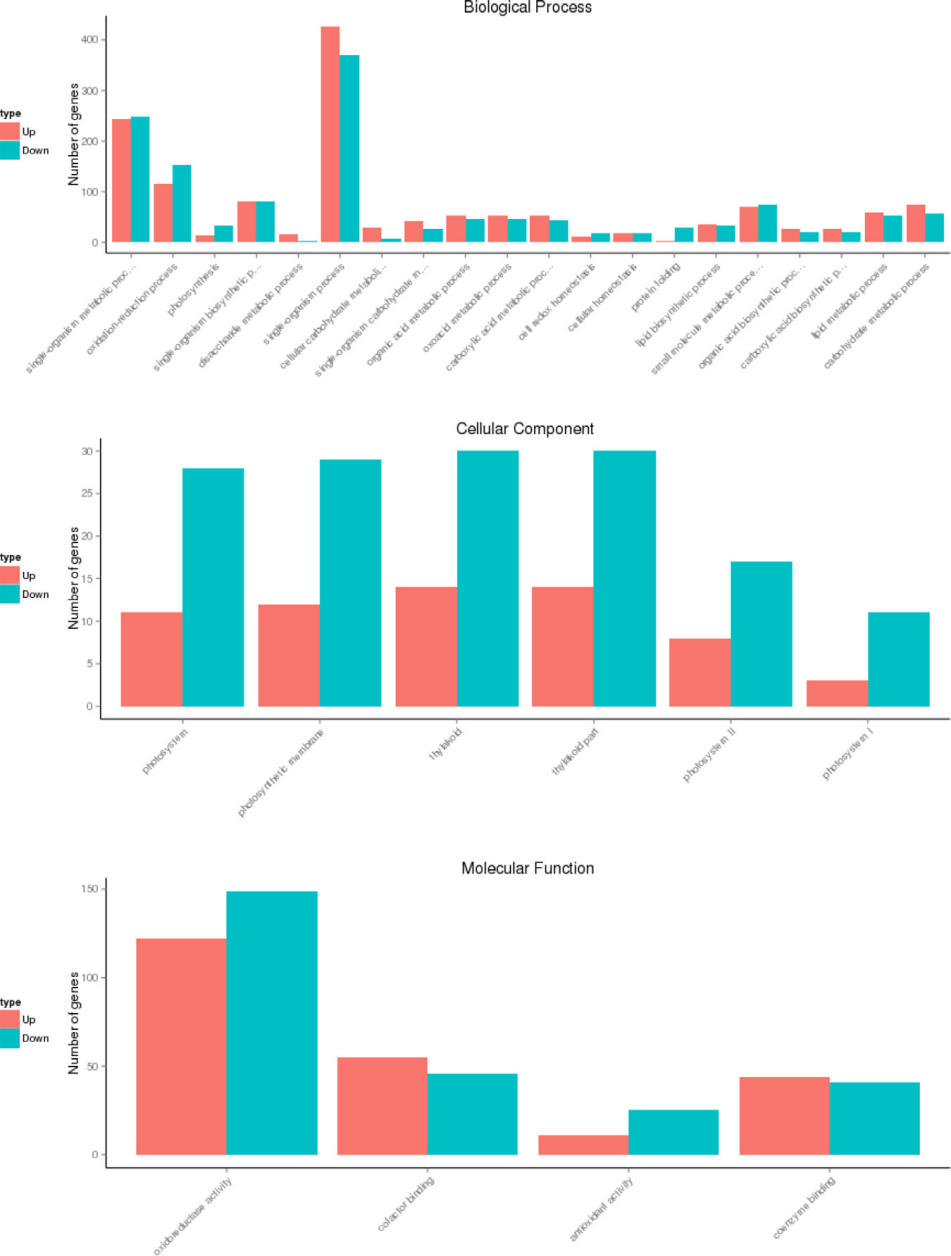
GO term enrichment analysis for DEG identified in RRIM 2025 Orange highlights the up-regulated genes and blue shows the down-regulated genes in RRIM 2025.

The dataset contains raw sequencing data obtained through the transcriptome sequencing of leaf samples of the rubber tree (*Hevea brasiliensis*). The data files were deposited at the NCBI SRA database under project accession no. PRJNA723431. Table 1 describes the raw reads-generated assembly and annotation information for *H. brasiliensis* healthy and *Oidium heveae*-infected transcriptomes. Table 2 provides the information on transcriptome mapping statistics for healthy and *Oidium heveae*-infected examples of two *H. brasiliensis* clones. Fig. 1 shows the differentially expressed genes (DEG) in three gene ontology (GO) categories, viz., biological process, cellular component and molecular function, in the *H. brasiliensis* RRIM 929 clone. Fig. 2 shows the DEG in three GO categories, viz., biological process, cellular component and molecular function, in the *H. brasiliensis* RRIM 2025 clone. Supplementary files containing the research data were uploaded to Mendeley Data under the DOI:10.17632/2mcfj7hw2y. Table S1and S2 show downregulated and upregulated genes in *H. brasiliensis* clone RRIM 929 infected with *O. hevea* respectively. Table S3 and S4 show downregulated and upregulated genes in *H. brasiliensis* clone RRIM 2025 infected with *O. hevea* respectively.

**Table 1:** Read and assembly statistics for healthy and *Oidium heveae*-infected *Hevea brasiliensis*.

Plant material	Healthy RRIM 2025	Healthy RRIM 929	Infected RRIM 2025	Infected RRIM 929
Total number of raw reads	96070286	92007684	93324564	93747858
Total number of clean reads	89811922	86321620	87318644	88038748
Total number of bases	13.5G	12.9G	13.1G	13.2G
GC content (%)	43.61	42.9	43.74	43.61
Clean reads Q20 (%)	96.91	96.91	96.76	96.86
Clean reads Q30 (%)	91.9	91.95	91.65	91.84

**Table 2:** Reads and statistics for transcriptome mapping to the *Hevea brasiliensis* reference genome

Sample name	Healthy RRIM 2025	Healthy RRIM 929	Infected RRIM 2025	Infected RRIM 929
Total reads	86321620	89811922	88038748	87318644
Total mapped	70341987 (81.49%)	75104386 (83.62%)	73606616 (83.61%)	73105993 (83.72%)
Multiple mapped	3253897 (3.77%)	4461927 (4.97%)	3542544 (4.02%)	3045360 (3.49%)
Uniquely mapped	67088090 (77.72%)	70642459 (78.66%)	70064072 (79.58%)	70060633 (80.24%)
Read-1	34068629 (39.47%)	35793858 (39.85%)	35609920 (40.45%)	35695494 (40.88%)
Read-2	33019461 (38.25%)	34848601 (38.8%)	34454152 (39.14%)	34365139 (39.36%)
Reads map to ‘+’	33563302 (38.88%)	35328855 (39.34%)	35036818 (39.8%)	35042848 (40.13%)
Reads map to ‘-’	33524788 (38.84%)	35313604 (39.32%)	35027254 (39.79%)	35017785 (40.1%)
Non-splice reads	42106249 (48.78%)	43334491 (48.25%)	43203998 (49.07%)	41334685 (47.34%)
Splice reads	24981841 (28.94%)	27307968 (30.41%)	26860074 (30.51%)	28725948 (32.9%)

## 2 Experimental Design, Materials and Methods

### 2.1 *Hevea brasiliensis* and *Oidium heveae*

*Hevea brasiliensis* clones RRIM 2025 and RRIM 929 were obtained from the Liman Plantation, Kedah. A spore suspension of *Oidium heveae* with 1.8 × 10^5^ spores per millilitre was prepared in 0.05% Tween 20 solution. The spore suspension was brushed onto the young leaves of test clones, which were kept at optimum infection conditions with high relative humidity above 85%, at a temperature range of 22–28°C, for a 16h light/8h dark photoperiod [[1], [2], [3]]. The leaves with the visible fungal growth of infected trees as well as those from healthy trees were harvested for RNA extraction.

### 2.2 Total RNA isolation and sequencing

Total RNA for RNA sequencing was isolated from one plant per treatment for each rubber tree clone. For the infected tree, RNA was isolated 21 days after infection. The RNA from the leaf samples was extracted using Plant RNA Reagent (Invitrogen, USA) according to the manufacturer's protocol after the tissue was homogenized using liquid nitrogen. Agarose gel electrophoresis was used to check for sharp 16S and 23S RNA bands to verify the integrity of the RNA samples. The Nanophotometer® spectrophotometer was used to verify RNA purity (IMPLEN, USA). The Qubit® RNA Assay Kit in the Qubit® 2.0 Fluorometer was used to measure the RNA concentration (Life Technologies, CA, USA). The Bioanalyzer 2100 system's RNA Nano 6000 Assay Kit was used to determine the integrity of the RNA (Agilent Technologies, CA, USA). The cDNA library preparation was generated using the NEBNext® Ultra™ RNA Library Prep Kit for Illumina® (NEB, USA), and clustering and sequencing was performed on the Illumina Hiseq 2000 platform.

### 2.3 RNA-Seq mapping

The Illumina sequencing generated 125 bp/150 bp paired-end reads in FASTQ format from two RRIM 2025 (healthy and infected) plants and two RRIM 929 (healthy and infected). The *Hevea brasiliensis* reference genome ASM165405v1 was used for the mapping-based transcriptome assembly of the RRIM 2025 and RRIM 929 data. The read quality assessment was performed using FastQC [4] to improve mapping accuracy. The Q30 score was above 91% for all samples. Tophat (v2.0.12) [5] successfully mapped the sequenced reads to the reference genome with 81% success. The transcript abundance was estimated using HTSeq (v0.6.1) software [6]. The expression normalization and differential expression was performed using DESeq2 (v1.12.0) [7].

The transcriptome data from healthy and Oidium heveae-infected rubber trees should provide information on the disease resistance potential of rubber clone RRIM 2025 and the disease susceptibility of rubber clone RRIM 925 as well as genes associated with disease resistance.

### 2.4 Accession code

The raw read data was submitted to Sequence Read Archive database (SRA) and can be accessed using the Data identification number PRJNA578136.

## Supplementary materials

Supplementary materials have been deposited to Mendeley Data and can be accessed through DOI:10.17632/2mcfj7hw2y

Table S1. List of genes downregulated in RRIM 929 in response to *O. heveae* infection.

Table S2. List of genes upregulated in RRIM 929 in response to *O. heveae* infection.

Table S3. List of genes downregulated in RRIM 2025 in response to *O. heveae* infection.

Table S4. List of genes upregulated in RRIM 2025 in response to *O. heveae* infection.

## Ethics Statement

This article does not contain any studies with human participants or animals performed by any of the authors.

## CRediT authorship contribution statement

**Urwashi Kamerkar:** Conceptualization, Methodology, Writing – review & editing, Supervision. **Ahmad Sofiman Othman:** Methodology, Software, Writing – original draft.

## Declaration of Competing Interest

The authors declare that they have no known competing financial interests or personal relationships which have, or could be perceived to have, influenced the work reported in this article.

## Data Availability

Transcriptome analysis of Oidium heveae infection on two Malaysian rubber clones (Hevea brasiliensis) (Original data) (NCBI).
